# Alternative reconstruction after pancreaticoduodenectomy

**DOI:** 10.1186/1477-7819-6-9

**Published:** 2008-01-28

**Authors:** Michael G Wayne, Irving A Jorge, Avram M Cooperman

**Affiliations:** 1 Department of Pancreatic and Biliary Surgery of New York, Cabrini Medical Center, New York, NY, USA

## Abstract

**Background:**

Pancreaticoduodenectomy is the procedure of choice for tumors of the head of the pancreas and periampulla. Despite advances in surgical technique and postoperative care, the procedure continues to carry a high morbidity rate. One of the most common morbidities is delayed gastric emptying with rates of 15%–40%. Following two prolonged cases of delayed gastric emptying, we altered our reconstruction to avoid this complication altogether. Subsequently, our patients underwent a classic pancreaticoduodenectomy with an undivided *Roux-en-Y *technique for reconstruction.

**Methods:**

We reviewed the charts of our last 13 Whipple procedures evaluating them for complications, specifically delayed gastric emptying. We compared the outcomes of those patients to a control group of 15 patients who underwent the Whipple procedure with standard reconstruction.

**Results:**

No instances of delayed gastric emptying occurred in patients who underwent an undivided *Roux-en-Y *technique for reconstruction. There was 1 wound infection (8%), 1 instance of pneumonia (8%), and 1 instance of bleeding from the gastrojejunal staple line (8%). There was no operative mortality.

**Conclusion:**

Use of the undivided *Roux-en-Y *technique for reconstruction following the Whipple procedure may decrease the incidence of delayed gastric emptying. In addition, it has the added benefit of eliminating bile reflux gastritis. Future randomized control trials are recommended to further evaluate the efficacy of the procedure.

## Background

Pancreaticoduodenectomy (Whipple procedure) is the standard treatment for operable adenocarcinomas of the head of the pancreas, as well as for other periampullary tumors and in some cases of chronic pancreatitis. One of the most common morbidities is delayed gastric emptying with rates of 15%–40% [1]. Advances in surgical skills and postoperative care have resulted in mortality rates of less than 5% [2]. The Whipple procedure involves resection of the head of the pancreas and the entire duodenum. The pancreas is reconstructed with a pancreaticojejunostomy, choledochojejunostomy, and gastrojejunostomy. The operation classically involves removal of the pylorus and antrum; however recently, surgeons have used a pylorus-preserving Whipple procedure to lower the incidence of postgastrectomy symptoms, such as delayed gastric emptying (DGE). Both methods – the standard and the pylorus-preserving Whipple – have their advocates, but each method continues to have gastroparesis as a postoperative problem.

Despite improvements in operative mortality rates, a high incidence of morbidity remains. Delayed gastric emptying is defined as the need for a nasogastric tube for 10 or more days or reinsertion of the tube owing to vomiting [3], and it is one the most common problems encountered postoperatively. Whether the standard and the pylorus-preserving Whipple is performed, it does not influence the rate of this complication, reported to be 20% to 40% [2].

In this cohort study, an undivided *Roux-en-Y *technique was used in our last 13 patients who underwent a Whipple procedure at our institution. No instances of delayed gastric emptying were observed. We then compared our study group with 15 patients receiving a Whipple procedure before the change in the method of reconstruction. We describe our operative technique and explain its technical aspects.

## Methods

This study took place at a 250-bed community hospital in Manhattan, New York, from January 2004 (at which time we changed our reconstruction technique) to October 2005. We have continued to use the new reconstruction technique owing to its excellent results.

During the study, the same team of surgeons used an undivided *Roux-en-Y *technique for reconstruction in 13 Whipple operations (5 women, 8 men; average age, 60.9 years; range, 47–79 years). There was no operative mortality, and there were no reoperations (Table 1). Table 2 lists the indications for the procedure. All of our patients were placed on proton pump inhibitors for 3 months. None of the patients received raglan^®^, erythromycin, or octreotide.

**Table 1:** Patient characteristics and risk factors

	**Undivided roux**	**Control**
Age	60.9 (47–79)	56.5 (45–68)
Sex		
Male	8	9
Female	5	6
Diabetes	7	8
Coronary Artery Disease	3	4
Peripheral Vascular Disease	2	3
Pancreatitis	2	4
Jaundice	9	11

**Table 2:** Indications for pancreatoduodenectomy

	**Undivided Roux**	**Control**
**Pathology**		
Adenocarcinoma	7 (54%)	9 (60%)
Ampullary	1 (8%)	0
Chronic Pancreatitis	2 (15%)	4 (27%)
Distal common bile duct cancer	2 (15%)	2 (13%)
Mucinous cystadenoma	1 (8%)	0

**Figure 1:**
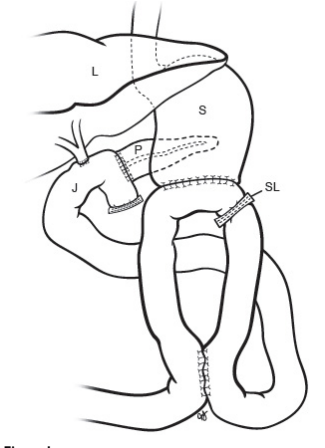
Reconstruction with uncut Roux-en-Y. P: pancreas, S: stomach, L: liver, J: jejunum, SL: staple line

We perform a classic Whipple resection with removal of the pylorus and antrum. A vagotomy is not performed. Reconstruction consists of a duct to mucosa pancreaticojejunostomy (end-to-side), then a duct to mucosa choledochojejunostomy (single layer). A 40 cm afferent limb is brought up through the ligament of Treitz, and an antecolic gastrojejunostomy is created with a GIA-75 stapler (Ethicon, Cornelia, Ga, USA). The ostomy made to apply the GIA is then closed in 2 layers, using 4-0 Vicryl on the inside layer and 3-0 silk on the outer layer (Figure 1). After this, the afferent limb is stapled closed with a TA-30 stapler (Ethicon, Cornelia, Ga, USA) just before it enters the stomach. We then measure 30 cm from the stomach, along the efferent limb, bringing up this section to the afferent limb where it is anastomosed. The anastomosis is created with a GIA-45 stapler (Ethicon, Cornelia, Ga, USA). The ostomy which was performed to apply the GIA is then closed in two layers as described previously.

## Results

There were no operative mortalities and no reoperations. The average operative time was 184 minutes. The average estimated blood loss was 230 mL. There were no cases of delayed gastric emptying with this technique. The mean duration of suction with a nasogastric tube was 24 hours. On average, patients were started on ice chips after 2 days. After 4 days, they were given 90 mL of clear liquids per meal. After 4 days, they were started on a standard clear liquid diet. The mean time to resumption of a regular diet was 8 days. The mean postoperative hospital stay was 8.4 days (range, 8–12 days). Complications are listed in Table 3. Average follow-up was 10 months and was possible in 13 patients. At follow-up, the patients denied nausea, vomiting, heartburn, abdominal pain, or postprandial bloating.

**Table 3:** Complications

	**Undivided Roux**	**Control**
**Complication**	**Incidence**	**Incidence**

Death	0	0
Reoperation	0	0
Delayed gastric emptying	0	2 (13%)
Wound infection	1 (8%)	2 (13%)
Pneumonia	1 (8%)	3 (20%)
Bleeding from gastrojejunal anastomosis	1 (8%)	0
Pancreatic fistula	0	1 (6%)
Intra-abdominal abscess	0	0

## Discussion

Delayed gastric emptying is a common complication after pancreaticoduodenectomy with rates ranging from 15%–40% [1]. Both the classic and the pylorus-preserving Whipple have this associated morbidity. It is a discouraging adverse event that is uncomfortable for the patients and increases their length of stay. As mentioned previously, delayed gastric emptying is defined as the need for a nasogastric tube for 10 or more days or reinsertion of the tube owing to vomiting. Utilizing the technique reported here, none of our patients experienced this complication. In our patients, the nasogastric tube was removed after 24 hours, at which time they were started on ice chips. On the fourth postoperative day, the patients were started on a limited clear liquid diet, and they advanced as they could tolerate. All patients were discharged on a regular diet 8 to 10 days postoperatively.

The exact mechanism for delayed gastric emptying after pancreaticoduodenectomy remains unclear. The most plausible cause might be due to disruption of the myoelectric activity of the gut [4]. One possible cause of DGE is removal of the cells in the duodenum that secrete motilin, which is a promotility agent. Another cause might be the irritating effect of bile on the gastric mucosa. It is because of these mechanisms that we changed our reconstruction technique after a Whipple procedure to an undivided *Roux-en-Y*. By utilizing this technique, the surgeon is not disrupting the myoelectric activity of the small bowel, as during a divided *Roux-en-Y*. The uncut *Roux-en-Y *permits propagation of myoneural transmission in the bowel wall, which also avoids development of ectopic pacemakers. These ectopic pacemakers, although functional, are much slower than our native duodenal pacemaker. Another advantage is that the stomach is not exposed to the irritating effects of bile, as it is in classic reconstruction, because the afferent limb is diverted into the efferent limb, and it is blocked off from the stomach. By diverting the intestinal contents and the pancreatic and biliary secretions away from the stomach, the surgeon protects the gastric mucosa from alkaline reflux [5-7].

## Conclusion

The uncut *Roux-en-Y *offers significant advantages over the classic Whipple reconstruction and pylorus-preserving reconstruction. Our technique has eliminated delayed gastric emptying. Although our sample size is small, there is a physiologic basis for this method. The deleterious effects of bile on the stomach are eliminated with this technique, and the surgeon maintains a myoneural bridge (which has been found to preserve the physiology of the small bowel). Future randomized control trials are recommended to further evaluate the efficacy of the procedure.

## Competing interests

The author(s) declare that they have no competing interests.

## Authors' contributions

**MW**: Creation of hypothesis and study design, co-surgeon involved in operations, **IJ**: Literature search and creation of discussion. Recompilation of patient data and analysis; **AC**: Description of technical procedure, co-study design and surgeon performing operations. All authors read and approved the final manuscript.
